# 

**Published:** 1883-02

**Authors:** 


					﻿VOL III.
FEBRUARY, 1883.
NO. 2.
THE
pOMIOPATHlC pHY^lClAJI,
A MONTHLY JOURNAL OF MEDICAL SCIENCE.
“ Magna est verilas, et prcevalebit."
HL CT. LEE. ZML ID.,
EDITOR.
CONTENTS:
(See inside Of Cover.)
PHILADELPHIA:
No. S1O9 CHESTNUT STRKKT.
□sr K "W T. O B K ;
A. L. CHATTERTON PUBLISHING COMPANY, Publisher’s Agents.
L.OJTT3O3SF ■■
ALFRED HEATH & CO., Sole Agents for Great Britain,
114 Ebury Street, Eaton Square.
Yearly Subscription, $2.50, invariably in advance.
Entered at the Philadelphia Post-Office as Second-Class Mail Matter.
				

